# Excessive Sleepiness and Longer Nighttime in Bed Increase the Risk of Cognitive Decline in Frail Elderly Subjects: The MAPT-Sleep Study

**DOI:** 10.3389/fnagi.2017.00312

**Published:** 2017-09-28

**Authors:** Audrey Gabelle, Laure-Anne Gutierrez, Isabelle Jaussent, Sophie Navucet, Caroline Grasselli, Karim Bennys, Cécilia Marelli, Renaud David, Sandrine Andrieu, Claudine Berr, Bruno Vellas, Yves Dauvilliers

**Affiliations:** ^1^Department of Neurology, Memory Research and Resources Center, CHU Montpellier, Montpellier, France; ^2^University of Montpellier, Montpellier, France; ^3^Institut National de la Santé et de la Recherche Médicale U 1183, Saint Eloi Hospital, Montpellier, France; ^4^Institut National de la Santé et de la Recherche Médicale U 1061, La Colombière Hospital, Montpellier, France; ^5^Department of Psychiatry, Memory Research and Resources Center, CHU Nice, Nice, France; ^6^Gérontopôle de Toulouse, Institut National de la Santé et de la Recherche Médicale UMR1027, Toulouse Université III, Toulouse, France; ^7^Department of Neurology, Narcolepsy National Reference Center, Sleep Center, CHU Montpellier, University of Montpellier, Montpellier, France

**Keywords:** sleep, Alzheimer disease, cognitive decline, frailty and cognitive impairment, excessive daytime sleepiness, amyloid

## Abstract

**Objective:** To identify self-reported sleep-wake disturbances that increase the risk of cognitive decline over 1-year follow-up in frail participants.

**Background:** Risk factors for cognitive impairment need to be better identified especially at earliest stages of the pathogenesis. Sleep-wake disturbances may be critical factors to consider and were thus being assessed in this at-risk population for cognitive decline.

**Methods:** Frail elderly participants aged ≥70 years were selected from a subsample of the Multi-domain Alzheimer Preventive Trial (MAPT) for a sleep assessment (MAPT-sleep study) at 18-month follow-up (M18). Sleep-wake disturbances were evaluated using a clinical interview (duration of daytime and nighttime sleep, time in bed, number of naps, and presence of clinically-defined sleep disorders) and numerous validated questionnaires [Epworth Sleepiness Scale for excessive daytime sleepiness (EDS), Insomnia Severity Scale and Berlin Questionnaire]. Cognitive decline was defined as a difference between the MMSE and cognitive composite scores at M24 and M36 that was ranked in the lowest decile. Multivariate logistic regression models adjusted for several potential confounding factors were performed.

**Results:** Among the 479 frail participants, 63 developed MMSE-cognitive decline and 50 cognitive composite score decrease between M24 and M36. Subjects with EDS had an increased risk of MMSE decline (OR = 2.46; 95% CI [1.28; 4.71], *p* = 0.007). A longer time spent in bed during night was associated with cognitive composite score decline (OR = 1.32 [1.03; 1.71], *p* = 0.03). These associations persisted when controlling for potential confounders. Patients with MMSE score decline and EDS had more naps, clinically-defined REM-sleep Behavior Disorder, fatigue and insomnia symptoms, while patients with cognitive composite score decline with longer time in bed had increased 24-h total sleep time duration but with higher wake time after onset.

**Conclusions:** The risk of cognitive decline is higher in frailty subjects with EDS and longer nighttime in bed. Early detection of sleep-wake disturbances might help identifying frail subjects at risk of cognitive decline to further propose sleep health strategies to prevent cognitive impairment.

http://www.clinicaltrials.gov NCT00672685; Date of registration May, 2nd 2008.

## Introduction

Age-related cognition and sleep-wake pattern changes vary considerably across individuals (Virta et al., 2013; Yaffe et al., 2014). Cross-sectional studies highlighted that poor sleep quality and excessive daytime sleepiness (EDS) are associated with cognitive impairment (Yaffe et al., 2007; Waller et al., 2016). Some longitudinal population-based and clinical-based cohort studies also confirmed these associations with disturbed nighttime sleep, shorter or longer nighttime sleep duration and EDS being all predictors of cognitive decline (Foley et al., 2001; Jaussent et al., 2012; Keage et al., 2012; Benito-Leon et al., 2013). However, other studies failed to validate some of these associations (Tworoger et al., 2006; Johar et al., 2016; Ramos et al., 2016). These contrasting findings could be explained by differences in the targeted populations (size, sex and age), follow-up period duration, cognitive tests, baseline cognitive status, sleep-wake assessment, sleep profile of participants, and control of potential confounders. Emerging evidence suggest the existence of a bidirectional role or at least a mechanistic interplay between disrupted sleep-wake cycle and Alzheimer's disease (AD) pathophysiology (Lucey and Holtzman, 2015; Cedernaes et al., 2017). Sleep-wake pattern alterations progressively increase with age, especially in elderly individuals with mild cognitive impairment (MCI), AD or at risk of AD (APOEε4 allele carriers) (Spira et al., 2013; Osorio et al., 2014; Sprecher et al., 2015; Branger et al., 2016; Drogos et al., 2016). Moreover, patients in early AD and in MCI cohorts often report sleep disorders such as sleep apnea syndrome (Emamian et al., 2016). Among cognitively normal elderly people, reduced and fragmented slow wave sleep has been associated with higher cerebrospinal fluid amyloid-β42 levels (Varga et al., 2016). However, only few studies prospectively confirmed using validated sleep questionnaires the presence of sleep disorders and determined precisely the spectrum of sleep-wake profiles at risk factor for cognitive impairment.

To our best knowledge, little is known about the potential relationship between cognitive decline and sleep profile in frail elderly populations. We wanted to assess both clinical and sleep profiles of frail elderly people whose cognitive performances declined over 1 year of follow-up. We hypothesized that among the frail elderly individuals enrolled in the Multi-domain Alzheimer Preventive Trial (MAPT), the presence of sleep-wake disturbances was independently associated with an increased risk of cognitive decline. To this aim, we evaluated the association between self-reported sleep-wake disturbances and cognitive status assessed with the Mini Mental State Examination (MMSE) test and a cognitive composite score in frail subjects aged 70 years and older over 1-year follow-up by taking into account many confounding factors.

## Materials and methods

### Participants

**Figure 1:**
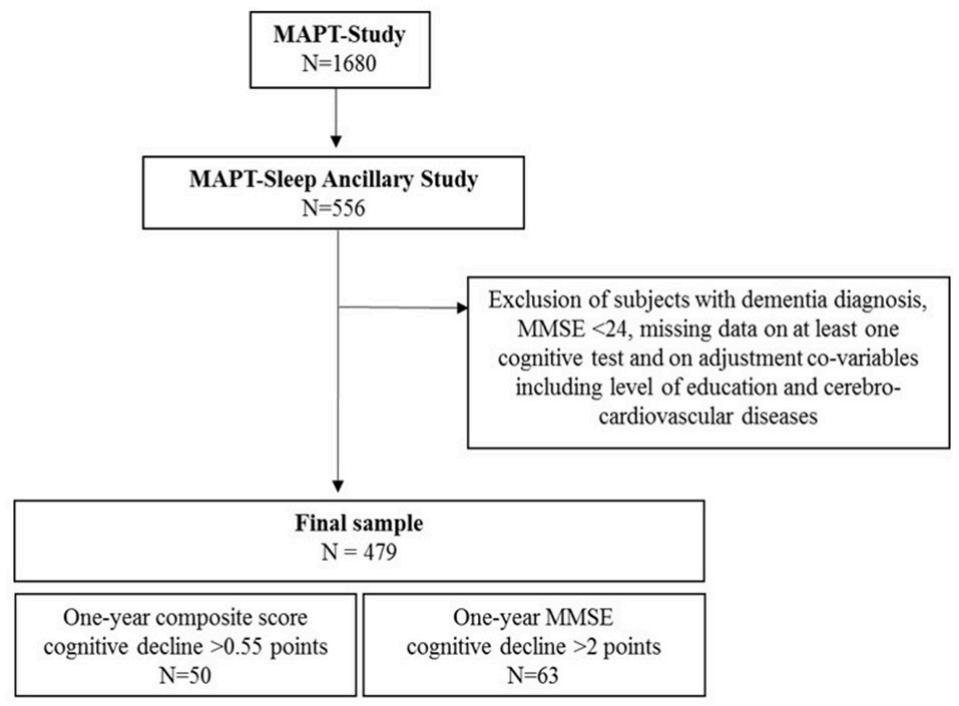
Study flow chart.

Participants for the sleep ancillary study were selected among the people enrolled in the MAPT (*n* = 1,680) (www.clinicaltrials.gov NCT00672685, date of registration May, 2nd 2008), a multi-center, randomized, placebo-controlled study designed to evaluate the efficacy of a multi-domain intervention (nutritional counseling, physical exercise and cognitive stimulation) in combination with omega-3 fatty acid supplementation in frail elderly individuals at risk of cognitive decline (Vellas et al., 2014). MAPT included ≥70-year-old community dwellers with good functional status and with at least one frailty criterion such as reporting spontaneous memory complaints to their primary care physician, limitation in one instrumental activity of daily living or slow walking gait (<0.8 m/s). Exclusion criteria were diagnosis of dementia (by a multidisciplinary committee based on the National Institute of Aging diagnostic criteria), baseline MMSE score <24/30, and/or dependency for one or more basic activities of daily living. Neuropsychological examination was carried out at baseline, and month 6 (M6), M12, M24, and M36 of the MAPT. Sleep assessment was proposed at M18 during a follow-up visit without neuropsychological assessment and 556 subjects agreed to complete the sleep evaluation and were included in the sleep ancillary study (Figure 1). To evaluate the association between sleep profile and cognitive status variations over 1 year, only subjects with cognitive evaluation at both M24 and M36 were finally retained. Moreover, participants who developed dementia or had MMSE < 24 between baseline and M24 and with missing data for at least one cognitive test or adjustment co-variables were also excluded. Finally, 479 subjects were selected for the sleep ancillary study. The MAPT and the sleep ancillary study were approved by the Toulouse ethics committee (CPP SOOM II, reference number 2-07-27). Written informed consent was obtained from all participants.

### Neuropsychological tests

For the sleep ancillary study, the MMSE score and the cognitive composite score (i.e., the MAPT primary cognitive outcomes) were used (Vellas et al., 2014). The MMSE was selected because it is routinely used in memory clinics. However, in frail individuals, cognitive decline may be subtle and the MMSE alone may not be precise enough to monitor changes in cognitive status. Therefore, a cognitive composite score was created. This score was calculated based on the z-scores of the 10-item MMSE orientation scale, Free and Cued Selective Reminding Test (FCSRT; sum of free recall and total recall), Digit Substitution Symbol Test (DSST) from the Wechsler Adult Intelligence Scale-revised (WAIS-R) and Category Naming Test (2 min). The mean and standard deviations of the baseline scores were used to calculate the z-score of each cognitive test. For this study, cognitive decline was defined as a difference between the M24 and M36 cognitive scores ranked in the lowest decile. Specifically, cognitive decline corresponded to a 2-point reduction of the MMSE total score and to a 0.55-point reduction of the cognitive composite score.

### Self-reported sleep parameters

With the help of the medical staff when required, participants were invited to complete sleep questionnaires and answer questions on their sleep-wake pattern such as duration of daytime and nighttime sleep, nighttime spent in bed (defined as the total time in bed with or without sleep during the night), presence and duration of naps (no nap, nap <1 h, nap ≥1 h). The medical staff assessed the presence of restless leg syndrome (RLS) based on the presence of the four minimal diagnostic criteria, and of possible rapid eye movement sleep behavioral disorder (RBD) clinically defined by the presence of violent nighttime agitation accompanied by shouting often at the end of the night and associated with dreamlike memories. The self-reported quantity of total sleep time was evaluated as the sum of daytime and nighttime sleep. The self-reported quality of sleep was measured as sleep efficiency (i.e., total sleep time divided by the time spent in bed, expressed as a percentage), and wake time after sleep onset (WASO). Sleep questionnaires included the Epworth Sleepiness Scale (ESS) to evaluate EDS with a cut-off score >10. Insomnia was assessed with the 7-item self-report Insomnia Severity Index (ISI) (score >7: low insomnia and score ≥15: moderate to severe insomnia). The risk of sleep apnea was evaluated with the Berlin Questionnaire that includes questions on daytime sleepiness, snoring, obesity and hypertension.

### Other clinical characteristics

The standardized interview included questions on sociodemographic characteristics, health status and medication use. Drugs were coded according to the World Health Organization Anatomical Therapeutic Chemical Classification. Hypnotics were classified as benzodiazepine (BZD), BZD-like compounds (zolpidem, zopiclone), and miscellaneous medications (including barbiturates, antihistamines, and other categories such as neuroleptics). Height and weight were measured to calculate the body mass index (BMI). Cerebro-cardiovascular and metabolic diseases were defined as self-reported history of stroke and cardiovascular events, diabetes, or hypertension (defined by a measured systolic blood pressure ≥140 mmHg or diastolic blood pressure ≥95 mmHg or current antihypertensive treatment). Depressive symptoms were evaluated using the Beck Depression Inventory (BDI) scale dichotomized in four validated clinical categories (0–11: no depressive symptoms; 12–19: mild symptoms; 20–27: moderate symptoms; 28–63: severe symptoms) and fatigue symptoms using the Chalder Fatigue Scale with scores reported in tertiles (1st: 0–4; 2nd–3rd: 5–14).

### Statistical analysis

The MMSE and cognitive composite scores were mostly skewed according to the Shapiro-Wilk test. Consequently, cognitive tests were categorized according to the cut-off described above. Associations between cognitive decline and sleep characteristics were analyzed using logistic regression models. The results were described with odds ratios (OR) and their 95% confidence intervals (CI). Randomization arm, sociodemographic and clinical variables associated with cognitive decline (*p* < 0.15) were included in the multivariate logistic regression models to estimate the adjusted ORs for sleep parameters. The *p*-value cut-off choice of 0.15 was based on the recommendations to use a significance level as high as 0.15 for variable selection in the univariate analysis as 0.05 can fail to identify variables known to be important. The assumption of linearity to the logit was tested for each continuous variables before their inclusion in the models and was significant. Participants with MMSE score reduction between M24 and M36 with and without EDS were compared using the Chi-2 or Fisher's exact test (discrete variables) and the Mann Whitney test (continuous variables). The same methodology was used to compare participants with cognitive composite score reduction between M24 and M36 with short and long nighttime spent in bed. For all analyses the significance level was set at *p* < 0.05. Analyses were performed using the SAS statistical software (version 9.4; SAS Inc., Cary, North Carolina).

## Results

### Baseline demographic and clinical characteristics

The median age of the 479 participants in the sleep ancillary study was 74 years [range: 70–88] with a predominance of women (67.85%) and intermediate education level (between 8 and 12 years of education). The APOEε4 allele was found in 21% of participants. Moreover, 41.5% were overweight (BMI [25–30]), 13.8% obese (BMI >30) and 62.6% reported cerebro-cardiovascular and/or metabolic disorders. The BDI score was higher than 11 in 28.0% of participants and the presence of fatigue symptoms found in 70.7% (score >5 at the Chalder Fatigue Scale).

### Evaluation of self-reported sleep profile

The risk of apnea evaluated clinically with the Berlin Questionnaire was detected in 40% of participants, RLS in 15.9%, clinically-defined RBD in 10.7%, and EDS (ESS score >10) in 20.6% including 3.8% with ESS >16. Moreover, 11.2% of participants had moderate to severe insomnia (ISI score ≥15), but less than 20% took hypnotic drugs. The median [min-max] self-estimated sleep duration was 7 h [3.5–10] for nighttime and 20 min [0–180] for daytime periods, although 12.1% had naps that lasted longer than 1 h. The median sleep efficiency was 87.5% [33–100]. Among participants with MMSE score decline between M24 and M36 (*n* = 63), 35.3% had EDS, fewer than 10% had moderate to severe insomnia (ISI score ≥15) and 8.1% RLS. Among participants with cognitive composite score decline between M24 and M36 (*n* = 50), 20% had EDS, 10% had moderate to severe insomnia (ISI score ≥15) and 14.3% had RLS.

### Risk factors of cognitive decline

The baseline demographic and clinical characteristics were not significantly different between participants with and without cognitive decline, based on the reduction of the MMSE score (*n* = 63) or the cognitive composite score (*n* = 50) between M24 and M36 (Table 1). Only fatigue symptoms were more frequently reported by participants with MMSE score reduction (*p* < 0.02). Age tended to be higher and cerebro-cardiovascular and metabolic disorders more frequent (*p* < 0.10) among participants with cognitive composite score decline compared to those without.

**Table 1:** Baseline sociodemographic and clinical characteristics of frailty participants according to MMSE cognitive decline and composite score cognitive decline over 1-year follow-up.

**Variable**	**MMSE-cognitive decliners**						**Cognitive composite** **score decliners**					
	**No**		**Yes**				**No**		**Yes**			
	***N*** = **416**		***N*** = **63**				***N*** = **429**		***N*** = **50**			
	***n***	**(%)**	***n***	**(%)**	**OR [95% CI]**	***Global p***	***n***	**(%)**	***n***	**(%)**	**OR [95% CI]**	***Global p***
Sex						0.17						
Women	287	68.99	39	60.32	1		291	67.83	34	68.00	1	0.98
Men	129	31.01	25	39.68	1.46 [0.85; 2.53]		138	32.17	16	32.00	0.99 [0.53; 1.86]	
Age, in years^a^	74 [70; 88]		74 [70; 87]		1.01 [0.95; 1.08]	0.77	74 [70; 88]		75 [70; 87]		1.07 [1.00; 1.14]	0.05
Level of education						0.20						0.27
Low	67	16.11	16	25.40	1		71	16.55	12	24.00	1	
Intermediate	201	48.32	27	42.86	0.56 [0.29; 1.11]		209	48.72	19	38.00	0.54 [0.25; 1.16]	
High	148	35.58	20	31.75	0.57 [0.28; 1.16]		149	34.73	19	38.00	0.75 [0.35; 1.64]	
APOE e4 allele						0.40						0.18
Not carrier	291	79.51	41	74.55	1		300	79.79	32	71.11	1	
Carrier	75	20.49	14	25.45	1.33 [0.69; 2.56]		76	20.21	13	28.89	1.60 [0.80; 3.20]	
Arm of randomization						0.13						0.68
Placebo	101	24.28	24	38.10	1		111	25.87			1	
Drug	107	25.72	11	17.46	0.43 [0.20; 0.93]		109	25.41		18.00	0.65 [0.27; 1.58]	
Placebo+Intervention	108	25.96	14	22.22	0.55 [0.27; 1.11]		107	24.94		30.00	1.11 [0.51; 2.41]	
Drug+Intervention	100	24.04	14	22.22	0.59 [0.29; 1.20]		102	23.78		24.00	0.93 [0.41; 2.11]	
BMI						0.68						0.67
Normal	188	45.41	25	39.68	1		192	44.96	21	42.00	1	
Overweight	170	41.06	28	44.44	1.24 [0.69; 2.21]		178	41.69	20	40.00	1.03 [0.54; 1.96]	
Obese	56	13.53	10	15.87	1.34 [0.61; 2.96]		57	13.35	9	18.00	1.44 [0.63; 3.33]	
Depression BDI scale						0.82						0.98
<12	279	72.09	41	70.69	1		290	71.96	30	71.43	1	
12–19	79	20.41	11	18.97	0.95 [0.47; 1.93]		81	20.10	9	21.43	1.07 [0.49; 2.35]	
20–27	21	5.43	5	8.62	1.62 [0.58; 4.53]		24	5.96	2	4.76	0.81 [0.18; 3.58]	
28–63	8	2.07	1	1.72	0.85 [0.10; 6.98]		8	1.99	1	2.38	1.21 [0.15; 9.99]	
Hypnotics intake						0.16						0.36
No	332	79.81	55	87.30	1		349	81.35	38	76.00	1	
Yes	84	20.19	8	12.70	0.57 [0.26; 1.25]		80	18.65	12	24.00	1.38 [0.69; 2.76]	
Fatigue scale						0.02						0.32
<5	108	27.37	25	42.97			123	30.00	10	22.73	1	
≥5	287	72.66	34	57.63	0.51 [0.29; 0.90]		287	70.00	34	77.27	1.46 [0.70; 3.04]	
CV and metabolic disorders						0.21						0.08
No	151	36.30	28	44.44	1		166	38.69	13	26.00	1	
Yes	265	63.70	35	55.56	0.71 [0.42; 1.22]		263	61.31	37	74.00	1.80 [0.93; 3.48]	
Obstructive chronic bronchitis						0.29						0.94
No	388	93.27	61	96.83	1		402	93.71	47	94.00	1	
Yes	28	6.73	2	3.17	0.45 [0.11; 1.96]		27	6.29	3	6.00	0.95 [0.28; 3.25]	
Cancer						0.70						0.87
No	294	70.67	46	73.02	1		304	70.86	36	72.00	1	
Yes	122	29.33	17	26.98	0.89 [0.49; 1.61]		125	29.14	14	28.00	0.95 [0.49; 1.81]	

a *Quantitative variables are expressed as median [minimum value; maximum value]*.

Participants with EDS had increased risk of MMSE score decline (*p* = 0.006). This risk remained significant after controlling for age, sex, education level, randomization arm, cerebro-cardiovascular and metabolic diseases (*p* = 0.007), and additional adjustments for the Chalder fatigue scale score and APOEε4 status (OR = 2.73 95% CI = [1.30; 5.72]; *p* = 0.008) (Table 2). No other sleep-wake disturbances were significantly associated with MMSE score decline in unadjusted and adjusted associations.

**Table 2:** Non-adjusted and adjusted associations between sleep complaints (a) of participants at baseline and cognitive decline (MMSE and Composite score) over 1-year follow-up.

	**MMSE-cognitive decliners**									**Cognitive composite** **score decliners**							
	***N*** = **416**		***N*** = **63**			**Model 0**		**Model 1**		***N*** = **429**		***N*** = **50**		**Model 0**		**Model 1**	
	***n***	**(%)**	***n***	**(%)**		**OR [95% CI]**	***Global P***	**OR [95% CI]**	***Global P***	***n***	**(%)**	***n***	**(%)**	**OR [95% CI]**	***Global P***	**OR [95% CI]**	***Global P***
**SLEEP PARAMETERS**																	
Night-time sleep, hour^a^	7 [3.5–10]		7 [3.5–10]			0.93 [0.75; 1.14]	0.47	0.89 [0.72; 1.11]	0.31	7 [3.5–10]		7 [3.5–10]		1.12 [0.88; 1.41]	0.36	1.12 [0.88; 1.41]	0.36
Day-time sleep, minute^a^	20 [0–180]		17.5 [0–180]			1.00 [0.99; 1.01]	0.66	1.00 [0.99; 1.01]	0.99	20 [0–180]		20 [0–120]		1.00 [0.99; 1.01]	0.68	1.00 [0.99; 1.01]	0.61
Naps							0.53		0.62						0.22		0.11
None	174	44.05	28	47.48		1		1		185	45.45	17	36.17	1		1	
Less 1 h	175	44.30	22	37.29		0.78 [0.43; 1.42]		0.78 [0.42; 1.44]		171	42.01	26	55.32	1.65 [0.87; 3.16]		1.81 [0.92; 3.53]	
More 1 h	46	11.65	9	15.25		1.22 [0.54; 2.76]		1.12 [0.49; 2.49]		51	12.53	4	8.51	0.85 [0.28; 2.65]		0.73 [0.23; 2.31]	
Total sleep night+day, hour^a^	7.5 [4–11]		7.38 [4–10.5]			0.91 [0.74; 1.12]	0.38	0.89 [0.72; 1.09]	0.26	7.5 [4–11]		7.7 [4.1–10.5]		1.07 [0.85; 1.35]	0.55	1.06 [0.85; 1.33]	0.60
Time in bed, hour^a^	8 [4–12.5]		8 [6–10]			0.88 [0.70; 1.10]	0.26	0.85 [0.67; 1.08]	0.18	8 [4–12.5]		8.5 [6–12]		1.34 [1.04; 1.71]	0.02	1.32 [1.03; 1.71]	0.03
Sleep efficiency,%^a^	87.5 [33–100]		87.5 [44–100]			1.00 [0.98; 1.02]	0.95	1.00 [0.98; 1.02]	0.83	87.5 [33–100]		87.5 [50–100]		0.99 [0.97; 1.01]	0.32	0.99 [0.97; 1.01]	0.39
WASO, hour^a^	1 [0; 8]		1 [0–4.5]			0.94 [0.73; 1.20]	0.61	0.94 [0.73; 1.23]	0.66	1 [0; 8]		1 [0–4.5]		1.16 [0.92; 1.46]	0.21	1.13 [0.89; 1.43]	0.33
EDS							0.006		0.007						0.92		0.96
<10	299	81.47	33		64.71	1		1		296	79.36	36	80.00	1		1	
>10	68	18.53	18		35.29	2.40 [1.28; 4.51]		2.46 [1.28; 4.71]		77	20.64	9	20.00	0.96 [0.44; 2.08]		0.98 [0.45; 2.16]	
Insomnia severity							0.35		0.47						0.45		0.38
< 8	215	52.44	39		61.90	1		1		223	52.72	31	62.00	1		1	
8–14	147	35.85	19		30.16	0.71 [0.40; 1.28]		0.76 [0.42; 1.39]		152	35.93	14	28.00	0.66 [0.34; 1.29]		0.64 [0.32; 1.25]	
≥15	48	11.71	5		7.94	0.57 [0.22; 1.53]		0.59 [0.22; 1.62]		48	11.35	5	10.00	0.75 [0.28; 2.03]		0.67 [0.24; 1.88]	
RLS							0.08		0.15						0.75		0.75
< 4 signs	341	82.97	57		91.94	1		1		356	83.96	42	85.71	1		1	
4 signs	70	17.03	5		8.06	0.43 [0.17; 1.10]		0.49 [0.19; 1.30]		68	16.04	7	14.29	0.87 [0.38; 2.02]		0.87 [0.37; 2.05]	
RBD							0.35		0.45						0.32		0.42
No	277	70.13	42		79.25	1		1		287	71.57	32	68.09	1		1	
Yes	43	10.89	5		9.43	0.77 [0.29; 2.05]		0.71 [0.26; 1.95]		40	9.98	8	17.02	1.79 [0.77; 4.17]		1.62 [0.68; 3.86]	
Sleep alone	75	18.99	6		11.32	0.53 [0.22; 1.29]		0.58 [0.23; 1.46]		74	18.45	7	14.89	0.85 [0.36; 2.00]		0.79 [0.33; 1.90]	
Risk of apnea							0.45		0.42						0.07		0.14
No	164	58.78	31		64.58	1		1		177	61.46	18	46.15	1		1	
Yes	115	41.22	17		35.42	0.78 [0.41; 1.48]		0.75 [0.38; 1.51]		111	38.54	21	53.85	1.86 [0.95; 3.65]		1.73 [0.83; 3.61]	

a *Quantitative variables are expressed as median [minimum value; maximum value]*.

*Model 0: Non-adjusted model*.

*Model 1: Model adjusted for age, sex, educational level, CV and metabolic disorders and arm of randomization*.

Participants with cognitive composite score reduction between M24 and M36 stayed longer in bed (>8.5 h) than those without (*p* = 0.02). This association remained significant after adjustment for potential confounders (*p* = 0.03) and also after additional adjustments for the Chalder fatigue scale score and APOEε4 status (OR = 1.40 95% CI = [1.04; 1.88]; *p* = 0.03) (Table 2). No further associations were found between cognitive composite score decline and other sleep-wake disturbances such as total sleep time or EDS.

### Self-reported sleep profile of frail patients with cognitive decline

Participants with MMSE score decline and EDS had more fatigue symptoms, slept longer during daytime, took more naps, had more insomnia and clinically-defined RBD than those without EDS (*p* < 0.05 for all these comparisons) (Table 3). Participants with cognitive composite score decline who spent longer time in bed (>8.5 h) reported increased nocturnal and 24-h total sleep time duration, but also higher WASO than those with shorter time in bed (*p* < 0.05 for all these comparisons).

**Table 3A:** Description of the clinical and sleep profile of MMSE score decliners.

***N***	**Modality**	**Among MMSE decliners**			**Among non MMSE decliners**		
		**No EDS**	**EDS**		**No EDS**	**EDS**	
		***N* = 33**	***N* = 18**	***p***	***N* = 299**	***N* = 68**	***p***
Age	(Years)	75 [70–85]	74 [70–87]	0.65^**^	74 [70–88]	73 [70–86]	0.55
Sex	Women	20 (60.6%)	10 (55.6%)	0.73	210 (70.2%)	41 (60.3%)	0.11
	Men	13 (39.4%)	8 (44.4%)		89 (29.8%)	27 (39.7%)	
BMI	Normal	14 (42.4%)	6 (33.3%)	0.53	134 (45.1%)	26 (38.2%)	0.30
	Overweight or obese	19 (57.6%)	12 (66.7%)	0.46	163 (54.9%)	42 (61.8%)	0.49
CV and metabolic diseases	Absence	13 (39.4%)	9 (50.0%)		110 (36.8%)	22 (32.3%)	
	Presence	20 (60.6%)	9 (50.0%)		189 (63.2%)	46 (67.6%)	
Hypnotics intake	No	29 (87.9%)	16 (88.9%)	0.99^*^	235 (78.6%)	58 (85.3%)	0.21
	Yes	4 (12.1%)	2 (11.1%)		64 (21.4%)	10 (14.7%)	
Fatigue scale	T1: [0–4]	18 (56.3%)	4 (22.2%)	**0.02**	91 (31.5%)	12 (17.9%)	**0.03**
	T2+T3: [5–14]	14 (43.8%)	14 (77.8%)		198 (68.5%)	55 (82.1%)	
Depression (BDI scale)	Non-depressed : <11	26 (81.25%)	9 (60.0%)	0.16^*^	208 (73.8%)	39 (59.09%)	**0.02**
	Depressed: >11	6 (18.75%)	6 (40.0%)		74 (26.2%)	27 (40.91%)	
Night-time sleep	(hour)	7 [5–9]	7 [3.5–10]	0.97^**^	7 [4–10]	7 [3.5–10]	0.49^**^
Day-time sleep	(hour)	10 [0–90]	35 [0–180]	0.006^**^	15 [0–120]	30 [0–180]	<0.0001^**^
Naps	Yes	11 (35.5%)	12 (70.6%)	**0.02**	149 (52.5%)	48 (75.0%)	**0.001**
	No	20 (64.5%)	5 (29.4%)		135 (47.5%)	16 (25.0%)	
Total sleep duration	Day+night	7.1 [5.0–9.5]	7.6 [4.2–10.5]	0.25^**^	7.5 [4.2–11.0]	7.7 [4.1–11.0]	0.39^**^
Time in bed	(Hour)	8 [6–9]	8 [6–10]	0.99^**^	8 [5–12.5]	8 [5–12]	0.42^**^
Sleep efficiency	(%)	87.5 [62.5–100]	88.2 [43.8–100]	0.80^**^	87.5 [33.3–100]	87.5 [53.8–100]	0.92^**^
WASO	(Hour)	1 [0–3]	1 [0–4.5]	0.45^**^	1 [0–8]	1 [0–4]	0.84^**^
Insomnia severity	<7	26 (78.8%)	8 (44.4%)	**0.013**	165 (55.6%)	26 (38.8%)	**0.013**
	>7	7 (21.2%)	10 (55.6%)		132 (44.4%)	41 (61.2%)	
RLS	4 signs	3 (9.4%)	1 (5.6%)	0.99^*^	46 (15.6%)	16 (23.5%)	0.12
	Less than 4 signs	29 (90.6%)	17 (94.4%)		248 (84.4%)	52 (76.5%)	
RBD	No	26 (96.7%)	9 (69.2%)	0.03^*^	201 (88.2%)	46 (83.6%)	0.37
	Yes	1 (3.7%)	4 (30.8%)		27 (11.8%)	9 (16.4%)	
Risk of apnea	No	16 (66.7%)	10 (58.8%)	0.61	122 (59.8%)	28 (54.9%)	0.52
	Yes	8 (33.3%)	7 (41.2%)		82 (40.2%)	23 (45.1%)	

* *Fisher exact test*.

** *Wilcoxon rank test*.

*The bold values mean that the result is significant with a p-value < 0.05*.

**Table 3B:** Description of the clinical and sleep profile of composite score decliners.

***N***	**Modality**	**Among composite score decliners**			**Among composite score non-decliners**		
		**Time in bed <8.5 h**	**Time in bed >8.5 h**	**P-Chi2**	**Time in bed < 8.5 h**	**Time in bed >8.5 h**	**P-Chi2**
		***N*** = **24**	***N*** = **25**		***N*** = **264**	***N*** = **160**	
Age	(Years)	75.0 [70–84]	74.0 [70–87]	0.53^**^	74.0 [70–88]	74.0 [70–88]	0.28^**^
Sex	Women	17 (70.8%)	16 (64.0%)	0.61	182 (68.9%)	106 (66.3%)	0.57
	Men	7 (29.2%)	9 (36.0%)		82 (31.1%)	54 (33.8%)	
BMI	Normal	10 (41.7%)	11 (44.0%)	0.87	124 (47.1%)	65 (40.9%)	0.21
	Overweight or obese	14 (58.3%)	14 (56.0%)		139 (52.9%)	94 (59.1%)	
CV and metabolic diseases	Absence	8 (33.3%)	5 (20.0%)	0.29	96 (36.4%)	67 (41.9%)	0.26
	Presence	16 (66.7%)	20 (80.0%)		168 (64.6%)	93 (58.1%)	
Hypnotics intake	No	20 (83.3%)	17 (68.0%)	0.21	221 (83.7%)	123 (76.9%)	0.08
	Yes	4 (16.7%)	8 (32.0%)		43 (16.3%)	37 (23.1%)	
Fatigue scale	T1: [0–4]	6 (28.6%)	3 (13.6%)	0.28^*^	71 (28.1%)	48 (31.6%)	0.45
	T2+T3: [5–14]	15 (71.4%)	19 (86.4%)		182 (71.9%)	104 (68.4%)	
Depression (BDI scale)	Non-depressed: <11	12 (66.7%)	17 (73.9%)	0.61	181 (73.0%)	105 (70.0%)	0.52
	Depressed: >11	6 (33.3%)	6 (26.1%)		67 (27.0%)	45 (30.0%)	
Night-time sleep	(Hour)	7 [3.5–8.25]	8 [5–10]	0.02^**^	6.5 [3.5–8]	8 [4–10]	<0.0001^**^
Day-time sleep	(Hour)	20 [0–60]	20.0 [0–120]	0.84^**^	15.0 [0–180]	20 [0–140]	0.13^**^
Naps	No	9 (40.9%)	8 (33.3%)	0.59	119 (48.0%)	63 (40.9%)	0.17
	Yes	13 (59.1%)	16 (66.7%)		129 (52.0%)	91 (59.1%)	
Total sleep	Day+night (hour)	7.1 [4.1–8.5]	8.3 [5.0–10.5]	0.009^**^	7.0 [4.0–11.0]	8.3 [4.0–11.0]	<0.0001^**^
Sleep efficiency	(%)	87.5 [50–100]	80 [52.6–100]	0.21^**^	87.5 [43.8–100]	88.9 [33.3–100]	0.04^**^
WASO	(Hour)	1 [0–4]	2 [0–4.5]	0.02^**^	1 [0–4.5]	1 [0–8]	<0.0001^**^
EDS	<10	18 (78.3%)	17 (80.9%)	0.99^*^	175 (78.1%)	117 (80.7%)	0.55
	>10	5 (21.7%)	4 (19.1%)		49 (21.9%)	28 (19.3%)	
Insomnia severity	<7	17 (70.8%)	13 (52.0%)	0.18	130 (49.8%)	88 (56.1%)	0.22
	>7	7 (29.2%)	12 (48.0%)		131 (50.2%)	69 (44.0%)	
RLS	4 signs	4 (17.4%)	3 (12.0%)	0.70^*^	47 (17.9%)	21 (13.3%)	0.21
	Less than 4 signs	19 (82.6%)	22 (88.0%)		215 (82.1%)	137 (86.7%)	
RBD	No	13 (81.2%)	19 (79.2%)	0.99^*^	162 (85.3%)	122 (91.7%)	**0.08**
	Yes	3 (18.8%)	5 (20.8%)		28 (14.7%)	11 (8.3%)	
Risk of apnea	No	9 (50.0%)	9 (42.9%)	0.66	105 (58.7%)	721(66.4%)	0.2
	Yes	9 (50.0%)	12 (57.1%)		74 (41.3%)	36 (33.6%)	

* *Fisher exact test*.

** *Wilcoxon rank test*.

*The bold values mean that the result is significant with a p-value < 0.05*.

## Discussion

This study highlights the association between self-reported sleep-wake disturbances and risk of cognitive decline in a cohort of frail elderly individuals, a targeted at-risk population to better understand how cognitively normal subjects may develop prodromal/preclinical AD. Frail participants with EDS or longer nighttime in bed had increased risk of cognitive decline (MMSE and cognitive composite scores decline, respectively) during the 1-year follow-up, independently of potential confounding factors (demographic variables, cerebro-cardiovascular and metabolic diseases, fatigue and APOEε4 status). The clinical sleep assessment and numerous validated sleep questionnaires used in this study allowed a comprehensive description of the sleep profile and related sleep-wake disturbances of this population. Participants with MMSE score decline and EDS had more naps, longer daytime sleep, more clinically-defined RBD, fatigue and insomnia symptoms. Participants with cognitive composite score decline and longer time in bed had longer 24-h and nighttime sleep duration but also higher frequency of WASO.

We found a relationship between EDS and MMSE score decline over 1 year in frail elderly individuals older than 70 years. Associations between EDS and increased risk of cognitive decline in community-dwelling elderly subjects have been already described (Jaussent et al., 2012; Keage et al., 2012) as also between EDS and cognitive impairment (Ohayon and Vecchierini, 2002), and EDS and MCI or dementia (Foley et al., 2001; Merlino et al., 2010). Other studies did not confirm these associations despite a large aged population included (Johar et al., 2016) or when comparing cognitively stable to impaired participants in late midlife (Waller et al., 2016). Recently, a community-based elderly study using a standardized assessment of daytime sleepiness and sleep duration and a neuropsychological battery of tests showed that EDS and longer sleep duration predict worse cognitive performances and decline in executive functions (Ramos et al., 2016). The discrepancies between all these studies could be partly explained by variations in the sleep profile and sleep-wake disturbances (e.g., presence of an underlying sleep disorder), sleep assessments (e.g., isolated questions, validated questionnaires or scales, objective measurement by sleep actigraphy or polysomnography), populations investigated (e.g., elderly controls, frail individuals, at risk or at different stages of AD), sample size, study design (e.g., cross-sectional vs. longitudinal), cognitive decline (e.g., global or specific neuropsychological tests considered as continuous variables or with cut-offs) and potential underlying confounding factors (e.g., depression, drug-free or not, medication type, cardiovascular disorders) (Jaussent et al., 2011, 2013; Blachier et al., 2012). Here, we found that 20% of subjects had EDS, as expected for this age group (Slater and Steier, 2012). EDS is a multifactorial symptom that may be triggered by sleep-disordered breathing, poor sleeping habits, cardiovascular diseases, obesity, drug intake or depression (Slater and Steier, 2012). As each of these factors could also increase the risk of cognitive impairment, we added these potential confounding factors in our different models. However, none modified the association strength. We also found that compared with participants without EDS, those with MMSE score decline and EDS slept longer during the day, had more naps but without any change on the total sleep time or nighttime sleep duration. In this group, EDS was not related to sleep deprivation, RLS, risk of apnea, hypnotic drugs intake or depression. However, these participants reported more clinically-defined RBD and fatigue and insomnia symptoms, thus strongly suggesting significant alterations in both sleep continuity and quality.

We also found a relationship between the risk of cognitive decline assessed by the composite cognitive score already validated in this frail elderly population (Donohue et al., 2014) and nighttime in bed, but not with nighttime or daytime sleep duration or sleep efficiency. Another study showed that cognitive decline assessed by MMSE score variation was worse in older adults with poor sleep quality after controlling for potential confounders (Niu et al., 2016). The question of whether sleep duration is linked to cognitive decline is still debated. Several prospective studies highlighted that either long (Benito-Leon et al., 2009; Loerbroks et al., 2010; Devore et al., 2014) or short (Tworoger et al., 2006; Sterniczuk et al., 2013) sleep durations were associated with a greater risk of cognitive impairment and dementia. The KORA-Age study recently showed that the risk of cognitive decline increased in subjects with prolonged sleep duration and particularly in those already cognitively impaired (Johar et al., 2016). In our study, participants with composite cognitive score reduction over 1 year and longer nighttime in bed had longer 24-h and nighttime sleep duration but also a higher WASO than subjects with time spent in bed <8.5 h. This result suggests that cognitive decline may be associated with both long sleep duration and disturbed nighttime sleep. Accordingly, we proposed that time spent in bed and the related cut-off of 8.5 h, never reported in the literature so far, may be considered as potential risk factors for cognitive decline.

Recent evidences from the general population suggested spontaneous fluctuations over time of sleep symptoms including EDS (Fernandez-Mendoza et al., 2015). Sleep-wake changes are often predicted by lifestyle and psychological factors that are potentially modifiable. We could ask whether EDS and nighttime duration in bed represent stable conditions in a frail population that might help to further individualize a homogeneous group of subjects at major risk for neurodegenerative disorders. Although sleep deprivation and fragmentation negatively affect neuropsychological performances, little is known about the mechanisms underlying the association between chronic sleep-wake alterations and cognitive decline. Recent data suggest that EDS and nighttime sleep alteration may share common neurobiological changes and play a role in AD pathophysiology, especially in amyloid β (Aβ) brain burden (Spira et al., 2013; Sprecher et al., 2015; Varga et al., 2016). In elderly subjects, extreme sleep duration, poor sleep quality and EDS were associated with increased brain Aβ deposits (Spira et al., 2013; Sprecher et al., 2015; Branger et al., 2016). Among the several neurotransmitters involved in the regulation of the sleep-wake pattern, orexin-A/hypocretin-1 which promotes wakefulness could directly modulate Aβ metabolism (Slats et al., 2013; Dauvilliers et al., 2014; Roh et al., 2014). Other biological mechanisms could be involved such as α-synuclein alterations (as suggested by the higher frequency of clinically-defined RBD in patients with reduced MMSE score and EDS), and hypothalamic-pituitary axis impairment with increased cortisol, oxidative stress, low-grade inflammation, and metabolic changes that could be linked to cerebrovascular damages and abnormal sleep-related Aβ clearance (Xie et al., 2013; Lucey and Holtzman, 2015). Further follow-up studies using sleep questionnaires but also objective sleep/wake measurements, amyloid PET scan and volumetric MRI assessment might help monitoring sleep profile changes, brain amyloid load and neuronal injury in frail elderly participants with cognitive decline.

The strengths of the study are the prospective design, the well-characterized population with frailty criteria at baseline, the use of a large neuropsychological test battery, the extensive clinical and self-reported sleep evaluations, and the large number of potential confounding factors. Even taken into account a wide range of risk factors for cognitive decline, the possibility remains that unmeasured confounders may explain part of the association observed. The present study also has some limitations. The assessment of the sleep-wake disturbances was based on clinical interviews and questionnaires completed by the patients (with the caregiver/clinical team's help when required) and did not include objective measurements such as actigraphy or polysomnography. Due to the self-reporting nature of the questionnaires, recall biases and lack of accuracy in the responses might have occurred, with potential misunderstandings. However, the use of questionnaires to detect individuals with poor sleep quality or who complain of sleep disturbances is particularly relevant in the routine clinical practice, easier and less expensive than polysomnography in a sleep laboratory. Moreover, we are fairly confident concerning the self-reported sleep data in frail subjects without cognitive troubles (MMSE >24). The design of the study cannot disentangle the discrepancies in the association between sleep-wake disturbances (namely EDS and longer time in bed) and cognitive decline whether assessed by the MMSE or the composite cognitive score. Finally, the short follow-up period (1 year) may not identify all subjects in whom cognitive impairment could have progressed to dementia.

## Conclusion

Our results indicate that EDS and longer nighttime in bed are associated with cognitive decline in frail patients, independently of major confounding factors. Our findings may suggest the importance of early detection of abnormal sleep-wake pattern in frail elderly participants at risk of neurodegenerative diseases to further propose sleep health strategies to prevent cognitive impairment.

## Members of the MAPT/DSA group

### MAPT study group

*Principal investigator*: Bruno Vellas (Toulouse); *Coordination*: Sophie Guyonnet; Project leader: Isabelle Carrié; *CRA*: Lauréane Brigitte; *Investigators*: Catherine Faisant, Françoise Lala, Julien Delrieu, Hélène Villars; *Psychologists*: Emeline Combrouze, Carole Badufle, Audrey Zueras; *Methodology, statistical analysis and data management*: Sandrine Andrieu, Christelle Cantet, Christophe Morin; *Multi-domain group*: Gabor Abellan Van Kan, Charlotte Dupuy, Yves Rolland (physical and nutritional components), Céline Caillaud, Pierre-Jean Ousset (cognitive component), Françoise Lala (preventive consultation), Bertrand Fougère (Toulouse). The cognitive component was designed in collaboration with Sherry Willis from the University of Seattle, and Sylvie Belleville, Brigitte Gilbert and Francine Fontaine from the University of Montreal.

*Co-investigators in the associated centers*: Jean-François Dartigues, Isabelle Marcet, Fleur Delva, Alexandra Foubert, Sandrine Cerda (Bordeaux); Marie-Noëlle-Cuffi, Corinne Costes (Castres); Olivier Rouaud, Patrick Manckoundia, Valérie Quipourt, Sophie Marilier, Evelyne Franon (Dijon); Lawrence Bories, Marie-Laure Pader, Marie-France Basset, Bruno Lapoujade, Valérie Faure, Michael Li Yung Tong, Christine Malick-Loiseau, Evelyne Cazaban-Campistron (Foix); Françoise Desclaux, Colette Blatge (Lavaur); Thierry Dantoine, Cécile Laubarie-Mouret, Isabelle Saulnier, Jean-Pierre Clément, Marie-Agnès Picat, Laurence Bernard-Bourzeix, Stéphanie Willebois, Iléana Désormais, Noëlle Cardinaud (Limoges); Marc Bonnefoy, Pierre Livet, Pascale Rebaudet, Claire Gédéon, Catherine Burdet, Flavien Terracol (Lyon), Alain Pesce, Stéphanie Roth, Sylvie Chaillou, Sandrine Louchart (Monaco); Kristelle Sudres, Nicolas Lebrun, Nadège Barro-Belaygues (Montauban); Jacques Touchon, Karim Bennys, Audrey Gabelle, Aurélia Romano, Lynda Touati, Cécilia Marelli, Cécile Pays, Patrice Douillet (Montpellier); Philippe Robert, Franck Le Duff, Claire Gervais, Sébastien Gonfrier (Nice); Yves Gasnier and Serge Bordes, Danièle Begorre, Christian Carpuat, Khaled Khales, Jean-François Lefebvre, Samira Misbah El Idrissi, Pierre Skolil, Jean-Pierre Salles (Tarbes).

*MRI group*: Carole Dufouil (Bordeaux), Stéphane Lehéricy, Marie Chupin, Jean-François Mangin, Ali Bouhayia (Paris); Michèle Allard (Bordeaux); Frédéric Ricolfi (Dijon); Dominique Dubois (Foix); Marie Paule Bonceour Martel (Limoges); François Cotton (Lyon); Alain Bonafé (Montpellier); Stéphane Chanalet (Nice); Françoise Hugon (Tarbes); Fabrice Bonneville, Christophe Cognard, François Chollet (Toulouse).

*PET scan group*: Pierre Payoux, Thierry Voisin, Julien Delrieu, Sophie Peiffer, Anne Hitzel, (Toulouse); Michèle Allard (Bordeaux); Michel Zanca (Montpellier); Jacques Monteil (Limoges); Jacques Darcourt (Nice).

*Medico-economic group*: Laurent Molinier, Hélène Derumeaux, Nadège Costa (Toulouse).

*Biological sample collection*: Christian Vincent, Bertrand Perret, Claire Vinel (Toulouse).

*Safety management*: Pascale Olivier-Abbal.

### DSA group

Sandrine Andrieu, Stéphanie Savy, Christelle Cantet, Nicola Coley.

## Declarations

### Ethics approval and consent to participate

The MAPT study and the sleep ancillary study were approved by the ethics committee in Toulouse (CPP SOOM II).

Written informed consent was obtained from all participants.

### Consent for publication

No copyright or license for publication. As authors we have full access to all the data; and have the right to publish any and all data separate and apart from any sponsor. All authors have read the manuscript. All authors and contributors have agreed to the conditions noted on the Authorship Agreement Form. There was no ghost writing.

### Availability of data and material

The data that support the findings of this study are available from MAPT/DSA, but restrictions apply to the availability of the data used under license for the current study, and that, therefore, are not publicly available. Data are, however, available from the authors upon reasonable request and with permission from MAPT/DSA.

## Author contributions

AG and YD: Data analysis and interpretation; study concept; study supervision; drafting/revising the manuscript. LG: Data acquisition; data analysis and interpretation; statistical analysis; drafting/revising the manuscript. IJ and CB: Data analysis and interpretation; statistical analysis; drafting/revising the manuscript. SN, CG, KB and CM: Data acquisition; revising the manuscript. RD: Drafting/revising the manuscript. SA and BV: Data interpretation; drafting/revising the manuscript.

### Conflict of interest statement

The authors declare that the research was conducted in the absence of any commercial or financial relationships that could be construed as a potential conflict of interest.
